# COVID-19 as a Research Dynamic Transformer: Emerging Cross-Disciplinary and National Characteristics

**DOI:** 10.3389/fdata.2021.631073

**Published:** 2021-07-26

**Authors:** Ryosuke L. Ohniwa, Joji Kijima, Mizuho Fukushige, Osamu Ohneda

**Affiliations:** ^1^ Faculty of Medicine, University of Tsukuba, Tsukuba, Japan; ^2^ Center for Biotechnology, National Taiwan University, Taipei, Taiwan; ^3^ Bureau of Global Initiatives, University of Tsukuba, Tsukuba, Japan

**Keywords:** COVID-19, coronavirus research, researcher dynamics, PubMed, scientometrics

## Abstract

The outbreak of the COVID-19 pandemic has had an unprecedented impact on humanity as well as research activities in life sciences and medicine. Between January and August 2020, the number of coronavirus-related scientific articles was roughly 50 times more than that of articles published in the entire year of 2019 in PubMed. It is necessary to better understand the dynamics of research on COVID-19, an emerging topic, and suggest ways to understand and improve the quality of research. We analyze the dynamics of coronavirus research before and after the outbreaks of SARS, MERS, and COVID-19 by examining all the published articles from the past 25 years in PubMed. We delineate research networks on coronaviruses as we identify experts’ background in terms of topics of previous research, affiliations, and international co-authorships. Two distinct dynamics of coronavirus research were found: 1) in the cases of regional pandemics, SARS and MERS, the scope of cross-disciplinary research remained between neighboring research areas; 2) in the case of the global pandemic, COVID-19, research activities have spread beyond neighboring disciplines with little transnational collaboration. Thus, COVID-19 has transformed the structure of research on coronaviruses as an emerging issue. Knowledge on COVID-19 is distributed across the widest range of disciplines, transforming research networks well beyond the field of medicine but within national boundaries. Given the unprecedented scale of COVID-19 and the nationalization of responses, the most likely way forward is to accumulate local knowledge with the awareness of transdisciplinary research dynamics.

## Introduction

COVID-19 [SARS-CoV-2 (severe acute respiratory syndrome coronavirus 2)]—since first reported in Wuhan, China, in December 2019—has spread around the globe with more than 172 million confirmed cases and more than three million deaths from December 2019 to June 2021(WHO 2021). Earlier in this century, the world was also plagued by the outbreaks of SARS [SARS-CoV (severe acute respiratory syndrome coronavirus)]—originating from Guangdong Province, China, in November 2002 (Rosling and Rosling, 2003;Xu et al., 2004)—and MERS [MERS-CoV (Middle East respiratory syndrome coronavirus)] that emerged in March 2012 (Hijawi et al., 2013;Cauchemez et al., 2014). In the 21st century, coronaviruses have become the root causes of emerging infectious diseases in the world (Guarner, 2020;Wang et al., 2020).

**FIGURE 1:**
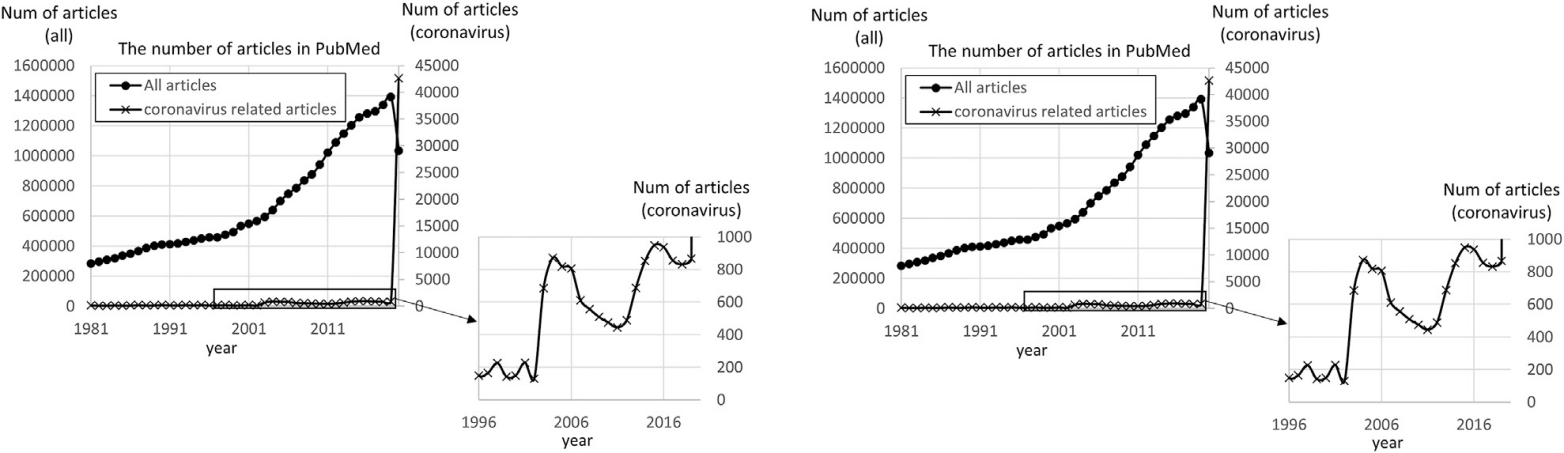
Number of articles on coronaviruses published in PubMed. The number of articles was counted on Aug 17th, 2020. “All articles” represents the total number of articles found in PubMed as published in each year (the scale is shown in the left *x* axis). “Coronavirus-related articles” represents the total number of articles found by the query of “coronavirus OR COVID-19 OR SARS-CoV OR MERS-CoV” (the scale is shown in the right *x* axis).

Meanwhile, the number of scientific research articles on coronaviruses in the fields of life sciences and medicine has increased dramatically (Figure 1). In the case of SARS-CoV, the number of publications increased fivefold by 2003—in just one year after the outbreak. In the case of MERS-CoV, the number increased steadily from the year after the outbreak in 2012 and doubled by 2015. In the case of SARS-CoV2, the number of research articles published between January 2020 and August 2020 was 50 times more than the number of scientific publications on coronaviruses in the entire year of 2019 (see Results). Indeed, coronaviruses have become an emerging research topic as a result (Rotolo et al., 2015).

It has been shown by bibliometric and sociological studies that researchers tend to publish articles on topics outside of their own area of expertise and begin diversifying their publications once certain topics—such as new technologies and concepts—are recognized as something scientifically valuable in the forthcoming future. That is how the corresponding topics become “emerging” (Van Merkerk and Van Lente, 2005;Borup et al., 2006;Gustafsson et al., 2015). At the same time, the rapid growth of publications makes these emerging topics the foci of cross-disciplinary studies (Rotolo et al., 2015). However, more studies are needed to adequately understand the relationship between emerging topics—such as infectious diseases—and the scope of cross-disciplinary research. This study aimed to fill part of that gap in the literature on the relationship between the emerging topics.

In this study, we analyze the Medical Subject Headings (MeSH) attached to PubMed (Medline) articles to identify the research topics and specialties of each researcher. PubMed (Medline), a literature database search engine run by the National Library of Medicine (NLM), contains approximately 10 million articles. MeSH is a popular keyword thesaurus developed by NLM, and it is typically used in PubMed to support literature searches (Lipscomb, 2000). It is attached to each article under the supervision of professional curators according to article contents (Lowe and Barnett, 1994). We have developed a method to identify emerging topics as clusters of emerging MeSH terms (Ohniwa et al., 2010;Ohniwa and Hibino, 2019). For the present study, we have modified this method to identify the characteristics of research topics and areas of expertise by selecting unique MeSH terms instead of those emerging (see Materials and Methods).

We investigate all the articles in PubMed between 1996 and 2020 to elucidate the following: 1) trends in coronavirus research before and after the outbreaks of the novel coronavirus infectious diseases—namely, SARS, MERS, and COVID-19—in terms of their impact on the nature of research; 2) the dynamics of how researchers venture across disciplinary boundaries to tackle emerging topics in times of crises by identifying their areas of research prior to the outbreaks and their countries of origin; and 3) the relational mechanism between cross-disciplinary research and transnational collaboration on coronaviruses. The results indicate the current COVID-19 pandemic has transformed coronavirus research into a nationalized body of knowledge across a wider range of disciplines. Finally, we suggest the most likely way forward for coronavirus research and draw an implication from the transformation of research dynamics based on the concepts of “transdisciplinarity (Stenner, 2017)” and “event (Whitehead, 1925)”.

## Materials and Methods

### Dataset

MeSH terms attached to articles published between 1996 and 2020 were collected through PubMed (https://www.ncbi.nlm.nih.gov/pubmed/) on August 17th, 2020. A total of 21,706,508 articles were included in the analysis.

MeSH terms attached on each article were identified from the XML data, and any overlaps in terms for each article were eliminated by our original Perl scripts as described in our previous articles (Ohniwa et al., 2010;Ohniwa and Hibino, 2019). Here, to identify the set of MeSH terms attached to each article, terms tagged as <DescriptorNameUI = @> and <NameOfSubstance UI = @> from the XML data (@ represents each UI) were extracted, and then overlaps in the terms for each article were eliminated by Perl with our original scripts. Then, to eliminate the terms not concerned with research topics, all the MeSH terms under the following hierarchies were excluded: “Geographicals [Z],”“Publication Characteristics [V],”“Named Groups [M],”“Health Care [N],”“Information Services [L01.453],”“Communications Media [L01.178],”“Communication [L01.143],”“Information Centers [L01.346],” and “Publishing [L01.737]” according to the 2018 MeSH Tree Hierarchy (https://meshb-prev.nlm.nih.gov/search). These categories involve MeSH terms which represent the style or the background of articles rather than its research contents. A total of 1,776,759 kinds of terms with a total of 957,790,657 occurrences were obtained between 1996 and 2020 by this operation.

To identify the sets of authors and affiliations attached to each article, terms tagged as <AffiliationInfo>, <LastName>, and <Initials> within <Author ValidYN = @> (@ represents each “Y or N”) were extracted by our original Perl script. A total of 42,923,027 kinds of authors (with affiliation) with a total of 56,467,393 occurrences were obtained by this operation (in the case of only author name, a total of 7,395,577 kinds of authors with a total of 108,977,113 occurrences).

Coronavirus-related articles published between 1996 and 2020 were separately obtained by searching PubMed using the query terms of “coronavirus OR COVID-19 OR SARS-CoV OR MERS-CoV.” A total of 56,077 articles and 66,850 kinds of MeSH terms with a total of 1,739,841 occurrences were obtained between 1996 and 2020 by this operation. From these articles, a total of 252,292 kinds of authors (with affiliations) with a total of 273,759 occurrences were obtained (in the case of using only authors’ names, a total of 157,593 kinds of authors with a total of 337,946 occurrences).

### Countries of Origin

The number of articles having the following words in the affiliation was counted: “Argentina”, “Australia”, “Austria”, “Belgium”, “Brazil”, “Bulgaria”, “Canada”, “Chile”, “China (‘China’ or ‘People’s Republic of China’)”, “Croatia”, “Czech Republic”, “Denmark”, “Egypt”, “Finland”, “France”, “Germany”, “Greece”, “HongKong”, “Hungary”, “India”, “Iran”, “Ireland”, “Israel”, “Italy”, “Japan”, “Korea”, “Malaysia”, “Mexico”, “Netherlands”, “New Zealand”, “Norway”, “Pakistan”, “Poland”, “Portugal”, “Romania”, “Russia”, “Saudi Arabia”, “Singapore”, “Slovakia”, “Slovenia”, “South Africa”, “Spain”, “Sweden”, “Switzerland”, “Taiwan (‘Taiwan’ or ‘Republic of China’)”, “Thailand”, “Turkey”, “Ukraine”, “United Arab Emirates (‘UAE’ or ‘United Arab Emirates’)”, “United Kingdom (‘England’, ‘U.K’, ‘UK’, ‘United Kingdom’, ‘Scotland’)”, and “United States (‘USA’ or ‘United States’)”.

### Unique Keywords

Among MeSH terms, we arbitrary defined unique keywords to coronavirus research as follows:

$$\left(A_{\alpha \;\mathrm{in}\;\beta }C/B_{\beta }\right)/\left(C_{\alpha \;\mathrm{in}\;\beta }/D_{\beta }\right)\geq 2,$$

where A_α in β_ is the number of appearances of the MeSH term α in years β found in coronavirus-related articles, B_β_ is the total number of the coronavirus-related articles in years β, C_α in β_ is the number of appearances of the MeSH term α in years β in PubMed, and D_β_ is the total number of articles counted in years β in PubMed. The terms whose rates were more than 2 were defined as unique keywords. A total of 13,125 kinds of MeSH terms as unique keywords with a total of 1,739,841 occurrences were collected between 1996 and 2020.

### Co-Word Analysis With Unique Keywords

Top 50 most frequently occurring unique keywords were collected for each period, and they were examined whether they coappeared in the same article. The coappearance was examined by using Perl with our original scripts. The coappearance of the keywords was visualized using Pajek software (Batagelj and Mrvar, 2002). To eliminate any weak relation among keywords, the threshold for making edges was set at 10% of the number of keywords (selecting smaller sized nodes) linked by the edges, according to the clusters appeared in the networks visualized by Pajek.

## Results

The number of articles covering coronaviruses in the fields of life sciences and medicine rapidly increased in 2003 and decreased until 2011, and it increased again in 2015 and decreased until 2018 (Figure 1). From Jan 1st, 2020 to Aug 17th, 2020, the number reached 42,647, which is approximately 50 times more than the number, 831 articles, in 2019. This tendency coincided with the emergence of coronavirus infectious diseases such as the emergence of SARS in November 2002 (Xu et al., 2004), MERS in November 2012 (Hijawi et al., 2013;Cauchemez et al., 2014), and COVID-19 in 2019. Once the diseases emerged, research on coronaviruses was rapidly activated and sustained for a few years. In the case of COVID-19, compared with SARS and MERS, the increment rate of the related articles was huge. Coronavirus has become an emerging research topic as a result (Rotolo et al., 2015).

### By Topic: Cross-Disciplinary Consequences and the Scope of Impact

Regarding their impact on research contents, this study identified unique keywords which represented the characteristics of coronavirus research before and after the outbreaks of SARS, MERS, and COVID-19. Since the collection of frequently appeared MeSH terms by itself did not reveal the unique characteristics of the research contents owing to their generality of use in numerous articles (Ohniwa et al., 2010), unique keywords from coronavirus research in a particular year were selected. These unique keywords defined as MeSH terms in coronavirus-related articles had the appearance rate that was at least twice as high as their appearance rate in all articles of the year (see Materials and Methods). This operation helped identify representative terms such as “SARS Virus” between 2003 and 2006 and “Middle East Respiratory Syndrome Coronavirus” between 2013 and 2016 as top 25 frequently appeared unique keywords (Table 1). Keywords related to “biological classification of coronaviruses,”“infection matters of coronaviruses,” and “respiratory tract issues [respiratory tract is the target infection site for coronaviruses as well as the site where its major symptom appears (Channappanavar and Perlman, 2017;Singhal, 2020)]” were commonly used between 1996 and 2020—consistent with the collection of unique keywords from coronavirus-related articles in this study. Keywords related to “components of the virus” and “biological aspect” were found in all of the years except for 2020, while others such as “public health” and “human” began to appear in 2020. When the list was expanded to top 50 frequently appeared unique keywords, this tendency was even strengthened (Supplementary Table S1). In the meantime, conventional keywords such as “immunology” were still found in all of the years. In addition to the fact that these top 50 keywords frequently coappeared in the coronavirus-related articles (Supplementary Figures S1–S6), the results of this investigation suggest the following: 1) the regional outbreaks of SARS and MERS did not change the cross-disciplinary research trends that had existed before the outbreaks as keywords fell well within the scope of existing well-connected networks for coronavirus research focusing on the topics which had been constantly studied regardless of the outbreaks; 2) the global outbreak of COVD-19, on the other hand, initially had a diversifying impact on the existing research trend as newly emerged keywords formed unconnected research networks across different disciplines, including areas of research such as jurisprudence and public policy.

**TABLE 1:** Top 25 unique keywords in coronavirus research articles.

	1996–2002	2003–2006	2007–2012	2013–2016	2017–2019	2020 Jan–Aug
Number of articles	1,189	3,180	3,083	3,427	2,551	42,647
Viruses	*1,113*	*2,646*	*2,631*	*2,705*	*1908*	15,166
RNA viruses	*1,100*	*2,611*	*2,547*	*2,596*	*1828*	15,154
Nidovirales	*1,058*	*2,533*	*2,349*	*2,422*	*1705*	15,129
Coronaviridae	*1,051*	*2,529*	*2,337*	*2,408*	*1,697*	15,129
Coronavirus	*1,046*	*2,508*	*2,311*	*2,336*	*1,682*	15,126
Proteins	*706*		*1742*		*910*	
Infections	*655*	*2092*	*1894*	*2,383*	*1707*	*17,959*
Virus diseases	*638*	*2041*	*1777*	*2,259*	*1,630*	*17,939*
RNA virus infections	*602*	*1960*	*1,608*	*2,103*	*1,506*	*17,929*
Nidovirales infections	*567*	*1901*	*1,473*	*1972*	*1,412*	*17,914*
Coronaviridae infections	*565*	*1899*	*1,463*	*1963*	*1,410*	*17,914*
Coronavirus infections	*551*	*1884*	*1,436*	*1912*	*1,406*	*17,912*
Cells	*534*		*1,039*			
Genetic phenomena	*518*		*1,214*		*708*	
Betacoronavirus	*506*	*1963*	*1,270*	*1,028*	*715*	14,995
Rodentia	*491*					
Muridae	*490*					
Murinae	*471*					
Mice	*459*		550			
Animal diseases	*446*	432	*838*	*1,006*	*898*	
Hepatitis viruses	*414*	212	301	132	59	
Biochemical phenomena	*412*		*1,035*			
Murine hepatitis virus	*408*	191	265	103	45	6
Cells, cultured	*379*	*625*	*784*	*478*	423	
Nucleic acids, nucleotides, and nucleosides	*369*	*704*	698	*494*	342	
Genetic techniques	352	*860*	*939*	*782*	*513*	805
Viral proteins	343	*913*	*1,064*	*705*	*502*	507
Genetic structures	333	*624*	599	442	290	
Nucleic acids	327	*590*	595	409	298	
Molecular structure	322	*732*	*737*	456	258	
Blood proteins	290	*570*	497	385	309	919
Artiodactyla	280		286	*601*	*603*	
Microbiological phenomena	264	412	680	*684*	*534*	734
Viral structural proteins	258	*653*	673	438	343	359
Alphacoronavirus	230	196	432	*525*	*539*	49
Virus physiological phenomena	189	345	534	*488*	354	481
Swine	160	67	174	375	*459*	
Respiratory tract infections	81	*1,591*	*866*	*659*	322	*17,774*
Disease outbreaks	39	404	199	389	277	*17,576*
Pneumonia	8	53	47	74	33	*17,732*
Pneumonia, viral	4	21	24	52	22	*17,719*
SARS virus	1	*1743*	*926*	301	115	576
Severe acute respiratory syndrome	1	*1,465*	569	230	77	393
Respiratory tract diseases		*1,617*	*911*	*696*	346	*17,785*
Amino acids, peptides, and proteins			*1756*		*930*	
Biological phenomena			542	*677*	*521*	
Middle East respiratory syndrome coronavirus				*625*	*500*	165
Health care quality, access, and evaluation					*587*	7,365
Eukaryota						*17,968*
Animals						*17,967*
Vertebrates						*17,947*
Chordata						*17,947*
Mammals						*17,939*
Eutheria						*17,938*
Primates						*17,845*
Haplorhini						*17,843*
Catarrhini						*17,842*
Hominidae						*17,813*
Humans						*17,812*
Environment and public health						*17,778*
Public health						*17,773*
Lung diseases						*17,741*

Italic numbers represent the number of appearances of keywords ranked in top 25. We sorted the order based on the total number in “1996–2002.”

### By Author: Identifying Converted Experts

The increase in the number of coronavirus-related articles after the outbreaks of SARS, MERS, and COVID-19 was largely due to the entry of new researchers (Supplementary Table S2). For example, between 2015 and 2019, a total of 24,745 authors with specific affiliations were identified in coronavirus-related articles. Their names with the same affiliations were found in only 369 out of 39,804 (0.9%) coronavirus-related articles in 2020. In the case of SARS, the authors of publications between 1998 and 2002 accounted for 23 out of 2,805 (0.8%) published articles on coronavirus between 2003 and 2006. In the case of MERS, the authors from the years between 2008 and 2012 occupied 123 out of 3,052 (4.0%) coronavirus-related articles published between 2013 and 2016. As a reference, we also analyzed the case of “Influenza,” resulting in a higher rate of occupation [the authors from the years between 2014 and 2018 occupied 1,364 out of 5,542 influenza-related articles published in 2019 (24.6%)]. Here, because not all affiliations were attached to the authors in PubMed before 2014 (Supplementary Table S3), the authors regardless of their affiliations were counted in (Supplementary Table S4). The risk of counting different authors as the same authors had to be taken. In any case, the results of this investigation show that most of these converted experts were conducting their coronavirus research on a temporary basis as they did not continue publishing on coronaviruses after new outbreaks.

This study then examined unique keywords used by the newcomers to coronavirus research from the past 5 years (searched by their names with affiliations). Those listed as top 25 frequently appeared unique keywords used by the newly entered researchers (Table 2) did not largely overlap with those of coronavirus research between 1996 and 2020 (Table 1) (11 out of 72 keywords). This tendency did not change when we compared them with top 50 frequently appeared unique keywords (Supplementary TablesS1, S5). Thus, it is likely that such new researchers came from different disciplines.

**TABLE 2:** Top 25 unique keywords used by newly entered researchers in the past.

MeSH	1998–2002 Author in 2003–2006	2008–2012 Author in 2013–2016	2015–2020 Author in 2020	MeSH	1998–2002 Author in 2003–2006	2008–2012 Author in 2013–2016	2015–2020 Author in 2020
Number of articles	70	681	49,798	Number of articles	70	681	49,798
Infections	*28*	*176*		History	5	*69*	
Genetic phenomena	*25*			Animal diseases	5	*46*	
Information science	*20*			History, Modern 1601-	4	*58*	
Bacterial infections and mycoses	*16*	*44*		Respiratory tract infections	3	*50*	*963*
Nucleic acids, nucleotides, and nucleosides	*16*			Orthomyxoviridae	3	*47*	373
Genetic structures	*16*			Influenza A virus	3	*47*	321
Genetic techniques	*16*			Influenza A virus	3	*47*	319
Nucleic acids	*16*			Organizations	3	41	*1,145*
Bacteria	*15*			Antiviral agents	3	15	*624*
Genome	*15*			Orthomyxoviridae infections	2	*50*	427
Biological science disciplines	*15*			Influenza, human	2	*42*	355
Natural science disciplines	*15*			United Kingdom	2		*1,265*
Viruses	*14*	*100*	*2079*	Social sciences		*162*	
Bacterial infections	*14*	*42*		Health care economics and organizations		*96*	
Molecular structure	*13*			Social control, formal		*70*	
Information services	*12*			Respiratory tract diseases		*64*	
Virus diseases	*11*	*126*	*2,903*	Medicine		*56*	
Immunologic techniques	*11*			Philosophy		*43*	
Genome components	*11*			Communications media		*43*	
Biology	*11*			Physicians		18	*756*
Blood proteins	*11*			Flaviviridae infections		7	*625*
Genes	*10*			Survival analysis			*1,658*
Sociology	*9*	*113*		Mortality			*1,522*
Social problems	*9*	41		Health planning			*1,436*
Warfare and armed conflicts	*9*	3		National health programs			*912*
Warfare	*9*	3		State medicine			*835*
Molecular sequence data	*9*			Survival rate			*774*
Computational biology	*9*			Urologic surgical procedures			*744*
Hemic and immune systems	*9*			Guidelines as topic			*678*
Proteobacteria	*9*			Controlled clinical trials as topic			*659*
Gram-negative bacteria	*9*			Randomized controlled trials as topic			*651*
Documentation	*9*			Registries			*647*
Base sequence	*9*			Radiotherapy			*641*
RNA viruses	8	*88*	*1,571*	Prostatic diseases			*594*
RNA virus infections	7	*110*	*2,261*	Practice guidelines as topic			*589*
Humanities	6	*141*		Proportional hazard models			*580*

Italic numbers represent the number of appearances of keywords ranked in top 25. We sorted the order based on the total number in “1998–2002 author in 2003–2006.”

**FIGURE 2:**
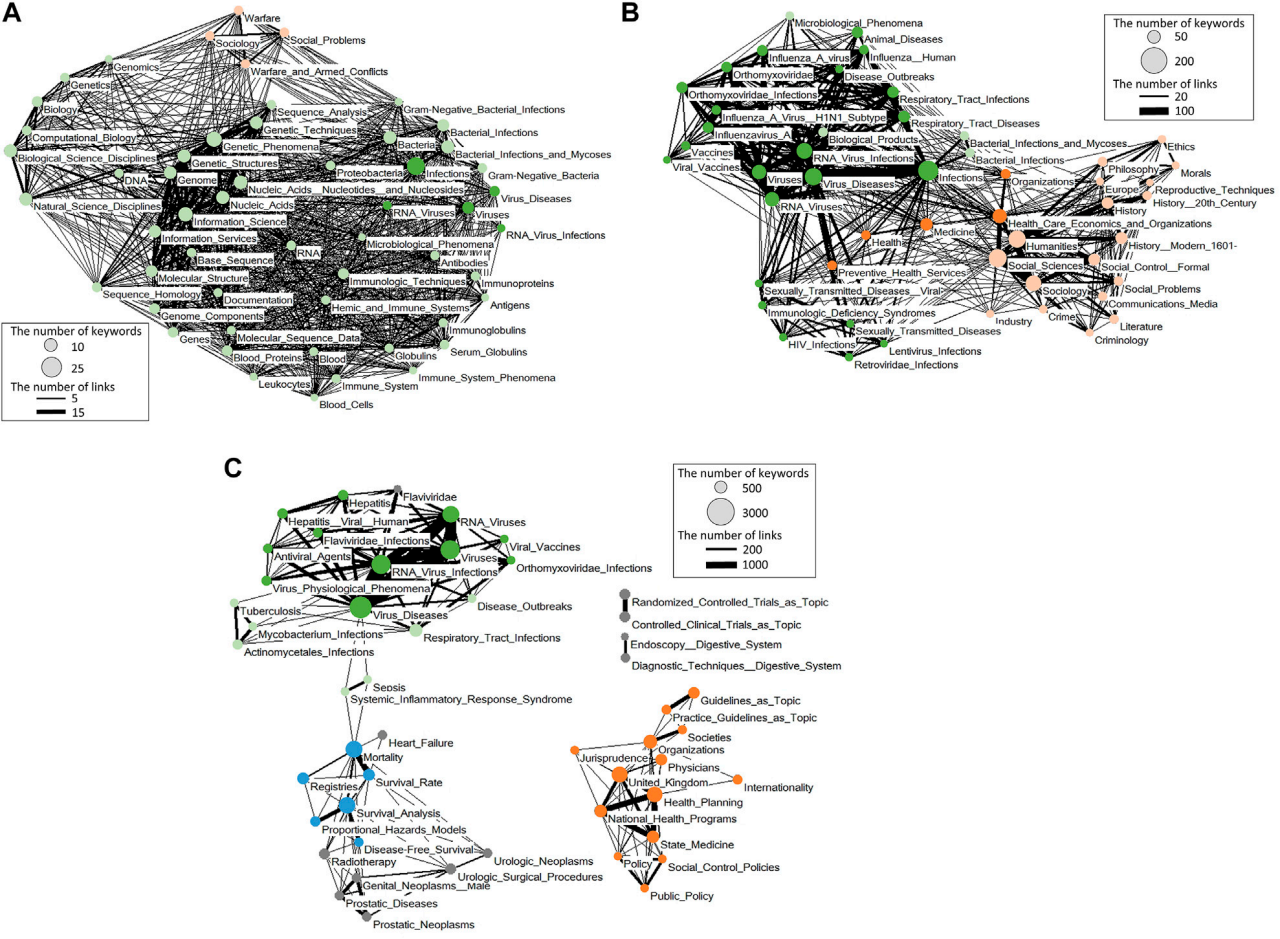
Networks of Top 50 unique keywords used by newcomers to coronavirus research. **(A)** The networks of top 50 unique keywords in 1998–2002, which were used by those researchers who converted to coronavirus research in 2003–2006. **(B)** The networks of top 50 unique keywords in 2008–2012, which were used by those who started coronavirus research in 2013–2016. **(C)** The networks of top 50 unique keywords in 2015–2019, which were used by those who converted to coronavirus research in 2020. The node colors represent the fields related to the keywords (green: virus and its infection; light green: molecular biology, microbiology, and immunology; orange: health care and policy; light orange: humanity and social issues; blue: epidemiology; gray: other issues). The threshold for making edges was set at 10% of the number of keywords (selecting smaller sized nodes) linked by the edges.

After the outbreak of SARS, experts on “infection of RNA viruses and bacteria especially in the fields of immunology, molecular biology, bioinformatics, and/or sociology” began to take part in coronavirus studies (Figure 2A). In the case of MERS, many RNA virus researchers handling “influenza virus infection” and “HIV infection” as well as experts on “health-care issues” joined in (Figure 2B). In the case of COVID-19, it attracted experts on the “hepatitis virus” and “*mycobacterium*” (Figure 2C).

Furthermore, for COVID-19, many converted experts had no background in the research on “RNA virus and infection.” They consequently formed a separate scientific network apart from those with their background in “RNA viruses and infections” in the beginning. They started from forming networks with those with their background in “urology and prostate,”“diagnosis of digestive system,”“clinical trial,” or “health care and guide line planning,”“jurisprudence,”“public policy,” and others. Such outside-in networking, due to the wide disciplinary base of the network, was one of the characteristics of COVID-19 research dynamics in the beginning.

### By Nationality: The Prioritization of National Contingencies as an International Trend

Once a novel infectious disease emerges in a certain country, scientific publications on the disease increase in the corresponding country (Table 3 and Supplementary Table S6). The United States and Germany have always been ranked among top 10 countries frequently publishing coronavirus articles, regardless of contingencies involving novel coronaviruses—suggesting that these countries have been the leaders of coronavirus research over the past 25 years. China became the No. 2 country after the SARS outbreak in November 2002 in China. Taiwan, Hong Kong, Singapore, and Canada—the countries and regions that prevailed after being hit hard by SARS in 2003 (Chan-Yeung and Xu, 2003; Wallinga and Teunis, 2004)—were also in top 10 between 2003 and 2006. In the case of the MERS outbreak in November 2012, Saudi Arabia was ranked among top 10 as one of the major countries that overcame the epidemic. Korea was also ranked among top 10 countries between 2013 and 2016, quite possibly due to the outbreak of the MERS epidemic in Korea in 2015 (Chen et al., 2017). With regard to the case of COVID-19, the exponential increase in the number of scientific publications originating from all the countries examined in this article suggests its impact on a global scale. It was also found that many researchers converted to the field of coronavirus studies after the outbreaks of infectious diseases (Supplementary Table S7). Accordingly, the outbreaks of novel coronavirus diseases accelerate coronavirus research activities in the affected countries by attracting new researchers.

**TABLE 3:** Top 10 countries for coronavirus articles in each period.

	1996–2002	2003–2006	2007–2012	2013–2016	2017–2019	2020 Jan–Aug
United States	1	1	1	1	2	1
Japan	2	8	4	7	6	
Canada	3	6	6		5	6
Germany	4	5	7	3	4	7
Spain	5		9			8
France	6	10		4	7	5
Italy	7	9	8		9	3
Switzerland	8					
Singapore	9	7				
Australia	10		10	9		9
Belgium	10					
United Kingdom	10					10
Korea				8	8	
Taiwan		4	5	10		
China		2	2	2	1	2
Hong Kong		3	3	6		
India						4
Saudi Arabia				5	3	
Egypt					10	

The value in each column represents the ranking for the number of articles published from the corresponding country in each period. We sorted the order based on the total number in “1996–2002.”

The proportion of internationally co-authored articles on coronaviruses to all the coronavirus-related articles among the countries listed in Supplementary Table S6 was 0.22–0.26 between 2016 and 2019 (Supplementary Table S8). This rate was higher than that of all the internationally co-authored articles during the same period (0.16–0.18). Thus, compared with the average collaboration ratio of articles, a higher rate of transnational collaboration was present for coronavirus research. In contrast, in 2020 after the outbreak of COVID-19, the rate of internationally co-authored coronavirus articles was 0.16. This was lower than the rate of all the international research articles published in the same year (0.19). In this way, transnational collaboration on coronavirus research as indicated in the rate of international co-authorships has decreased since the outbreak of COVID-19.

## Discussion

This study investigated the dynamics of research on COVID-19 (SARS-CoV-2) by comparing it to the previous cases of SARS (SARS-CoV) and MERS (MERS-CoV). Results show two different modes of research dynamics with regard to the scale of social impact as follows:1) in the cases of two regional pandemics, SARS and MERS, the scope of cross-disciplinary research remained between neighboring research areas as experts on surrounding research areas joined the networks of coronavirus experts; 2) in the case of the global pandemic, COVID-19, with overwhelming global impact, cross-disciplinary activities have spread far beyond neighboring areas of research to form new research networks. These dynamics of cross-disciplinary research are national in character as newly converted researchers came from the countries seriously affected by the coronaviruses. However, the majority of these converted experts are likely to conduct their coronavirus research on a temporary basis, and they might change their research subjects once an outbreak is over. Such temporary participation of researchers in coronavirus research suggests that securing the source of funding could be one of the factors for sustaining transdisciplinary research on coronaviruses and lowering the fatality of future outbreaks.

Knowledge on COVID-19 is distributed across a wide range of disciplines forming research networks within national boundaries. More technically, while MeSH terms may not be sufficient to completely identify the background of authors with different roles, this research shows that MeSH terms are still useful to the extent that they help identify the scope of cross-disciplinarity regarding coronavirus research. It would also be valuable to examine the dynamics of research beyond those fields covered by PubMed to further unveil the impact of COVID-19 on an even wider range of research activities in the world.

In short, COVID-19 has transformed the structure of coronavirus research. The greater the scale of social impact is, the more cross-disciplinary research emerges. In the case of COVID-19, the national character of research has been reinforced by the finding that transnational collaboration in terms of international co-authorships has decreased since the outbreak of the global pandemic. Given the unprecedented scale of COVID-19 and the nationalization of responses, the most likely way forward for medical experts is to accumulate local knowledge with the awareness of transdisciplinary research dynamics. For a coordinated response to COVID-19, an implication here is to be aware of the perspective that the global pandemic can be grasped into a bodily event for each medical and nonmedical expert to become an extension to a transdisciplinary solution to the health problem of the one and the many. An “event” or a “prehension” (apprehension which may or may not be cognitive) of things, here in this place such as a local response to the COVID-19 pandemic, has reference to other places since things gathered into the grasped unity of an event as a spatiotemporal unity here and now have essential reference to other places and other times (Whitehead, 1925). Events are prehensions of things that constitute realities of nature given that nature is a structure of evolving “processes” and each single event within its own context has all the reality that is interlocked with the whole (Whitehead, 1925). Finally, while the present study—with its focus on the articles published by August 17, 2020—demonstrated the initial impact of COVID-19, continuous research is still necessary to grasp the further transformation of research dynamics in the long-term challenge against COVID-19.

## Data Availability

The original contributions presented in the study are included in the article/Supplementary Material; further inquiries can be directed to the corresponding author.

## References

[B1] Batagelj V. Mrvar A. (2002). Pajek- Analysis and Visualization of Large Networks. Graph Drawing 2265, 477–478. 10.1007/3-540-45848-4_54

[B2] Borup M. Brown N. Konrad K. Van Lente H. (2006). The Sociology of Expectations in Science and Technology. Technol. Anal.Strateg.Manage. 18, 285–298. 10.1080/09537320600777002

[B3] Cauchemez S. Fraser C. Van Kerkhove M. D. Donnelly C. A. Riley S. Rambaut A. (2014). Middle East Respiratory Syndrome Coronavirus: Quantification of the Extent of the Epidemic, Surveillance Biases, and Transmissibility. Lancet Infect. Dis. 14, 50–56. 10.1016/s1473-3099(13)70304-9 24239323 PMC3895322

[B4] Chan-Yeung M. Xu R.-H. (2003). SARS: Epidemiology. Respirology 8 (Suppl. l), S9–S14. 10.1046/j.1440-1843.2003.00518.x 15018127 PMC7169193

[B5] Channappanavar R. Perlman S. (2017). Pathogenic Human Coronavirus Infections: Causes and Consequences of Cytokine Storm and Immunopathology. Semin.Immunopathol 39, 529–539. 10.1007/s00281-017-0629-x 28466096 PMC7079893

[B6] Chen X. Chughtai A.A. Dyda A. Macintyre C.R. (2017). Comparative Epidemiology of Middle East Respiratory Syndrome Coronavirus (MERS-CoV) in Saudi Arabia and South Korea. Emerg. Microbes Infect. 6, e51. 10.1038/emi.2017.40 28588290 PMC5520315

[B7] Guarner J. (2020). Three Emerging Coronaviruses in Two Decades. Am. J.Clin.Pathol. 153, 420–421. 10.1093/ajcp/aqaa029 32053148 PMC7109697

[B8] Gustafsson R. Kuusi O. Meyer M. (2015). Examining Open-Endedness of Expectations in Emerging Technological fields: The Case of Cellulosic Ethanol. Technol. Forecast.Soc. Change 91, 179–193. 10.1016/j.techfore.2014.02.008

[B9] Hijawi B. Abdallat M. Sayaydeh A. Alqasrawi S. Haddadin A. Jaarour N. (2013). Novel Coronavirus Infections in Jordan, April 2012: Epidemiological Findings from a Retrospective Investigation. East.Mediterr. Health J. 19 (Suppl. 1), S12–S18. 10.26719/2013.19.supp1.s12 23888790

[B15] Lipscomb C. E. (2000). Medical Subject Headings (MeSH). Bull Med. Libr. Assoc. 88, 265–266. 10928714 PMC35238

[B10] Lowe H. J. Barnett G.O. (1994). Understanding and Using the Medical Subject Headings (MeSH) Vocabulary to Perform Literature Searches. Jama-Journal Am. Med. Assoc. 271, 1103–1108. 10.1001/jama.271.14.1103 8151853

[B11] Ohniwa R. L. Hibino A. (2019). Generating Process of Emerging Topics in the Life Sciences. Scientometrics 121, 1549–1561. 10.1007/s11192-019-03248-z

[B12] Ohniwa R. L. Hibino A. Takeyasu K. (2010). Trends in Research Foci in Life Science fields over the Last 30 Years Monitored by Emerging Topics. Scientometrics 85, 111–127. 10.1007/s11192-010-0252-2

[B13] Rosling L. Rosling M. (2003). Pneumonia Causes Panic in Guangdong Province. BMJ 326, 416. 10.1136/bmj.326.7386.416 PMC1125313 12595377

[B14] Rotolo D. Hicks D. Martin B. R. (2015). What Is an Emerging Technology?. Res.Pol. 44, 1827–1843. 10.1016/j.respol.2015.06.006

[B16] Singhal T. (2020). A Review of Coronavirus Disease-2019 (COVID-19). Indian J.Pediatr. 87, 281–286. 10.1007/s12098-020-03263-6 32166607 PMC7090728

[B17] Stenner P. (2017). “Liminality and Experience A Transdisciplinary Approach to the Psychosocial, (London: Palgrave Macmillan UK Imprint). 10.1057/978-1-137-27211-9

[B18] Van Merkerk R. O. Van Lente H. (2005). Tracing Emerging Irreversibilities in Emerging Technologies: The Case of Nanotubes. Technol. Forecast.Soc. Change 72, 1094–1111. 10.1016/j.techfore.2004.10.003

[B19] Wallinga J. Teunis P. (2004). Different Epidemic Curves for Severe Acute Respiratory Syndrome Reveal Similar Impacts of Control Measures. Am. J.Epidemiol. 160, 509–516. 10.1093/aje/kwh255 15353409 PMC7110200

[B20] Wang Y. Wang Y. Chen Y. Qin Q. (2020). Unique Epidemiological and Clinical Features of the Emerging 2019 Novel Coronavirus Pneumonia (COVID‐19) Implicate Special Control Measures. J. Med.Virol. 92, 568–576. 10.1002/jmv.25748 32134116 PMC7228347

[B21] Whitehead A. N. (1925). Science and the Modern World Lowell Lectures, 1925. New York: The Macmillan company.

[B22] Xu R.-H. He J.-F. Evans M. R. Peng G.-W. Field H. E. Yu D.-W. (2004). Epidemiologic Clues to SARS Origin in China. Emerg. Infect. Dis. 10, 1030–1037. 10.3201/eid1006.030852 15207054 PMC3323155

