# Supplementary material for: COVID-19 as a Research Dynamic Transformer: Emerging Cross-Disciplinary and National Characteristics
Source: Front Big Data. 2021 Jul 26;4:631073. doi: 10.3389/fdata.2021.631073 (PMC8350321; doi:10.3389/fdata.2021.631073)
Supplement: Supplementary file 2 [file Image1.pdf]

(A)

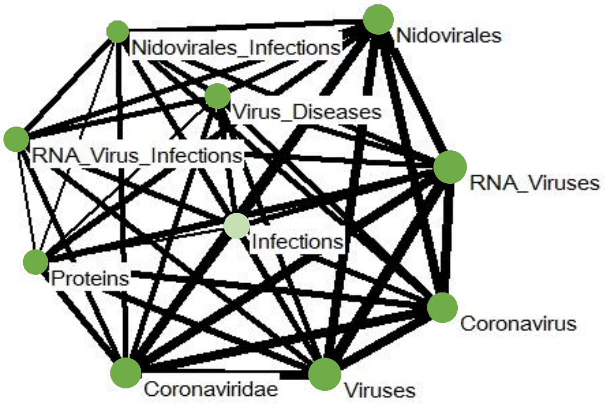

(B)

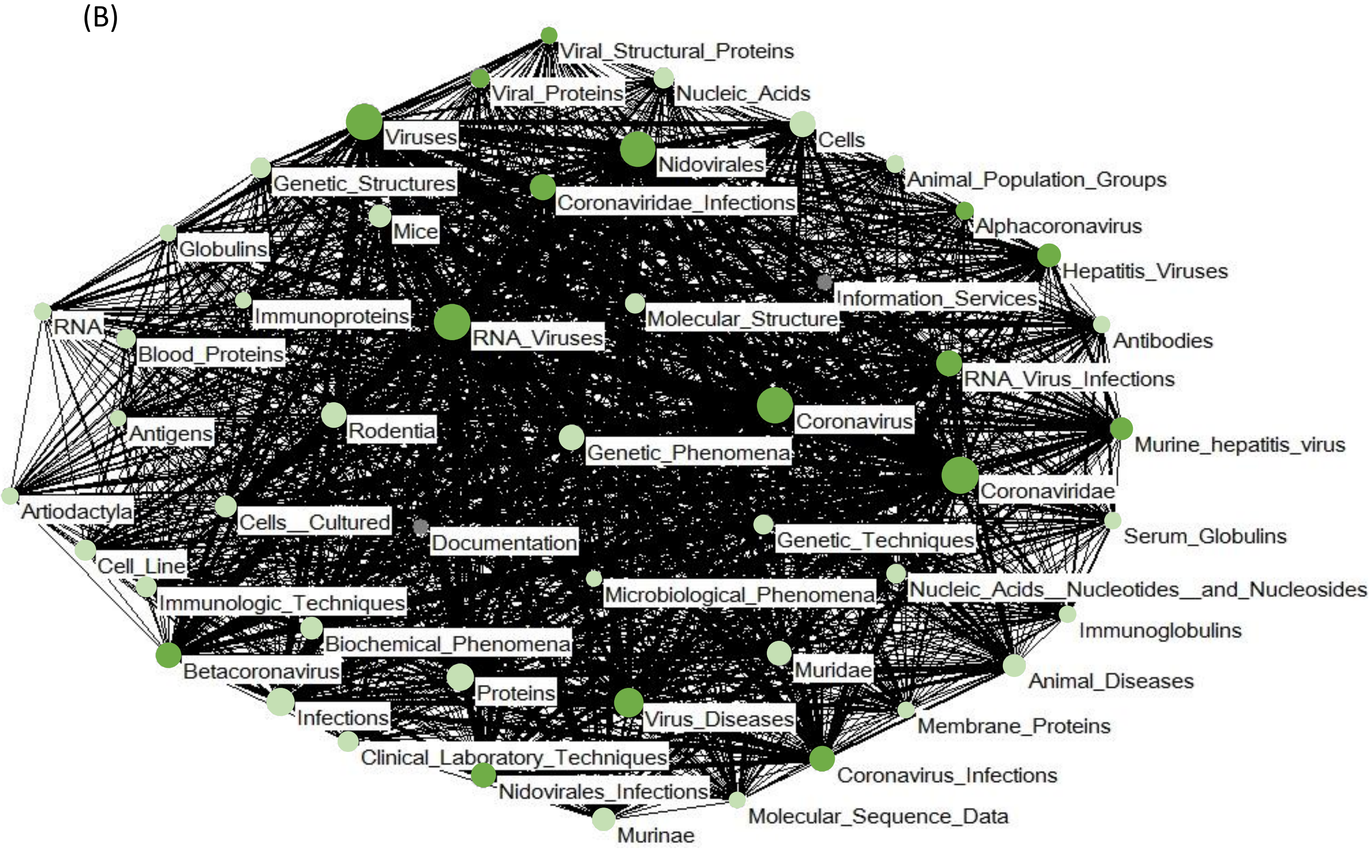

Supplementary Figure 1. The networks of the (A) top 10 and (B) top 50 unique keywords in 1996-2002. Only keywords that obtain links with other keywords are shown. The threshold for making edges was set at 10% of the number of keywords (selecting smaller sized nodes) linked by the edges. The node colors represent the fields related to the keywords (Green: virus and its infection, Light green: molecular biology, microbiology and immunology, Orange: health care and policy, Light orange: humanity and social issues, Blue: epidemiology, Grey: other issues).

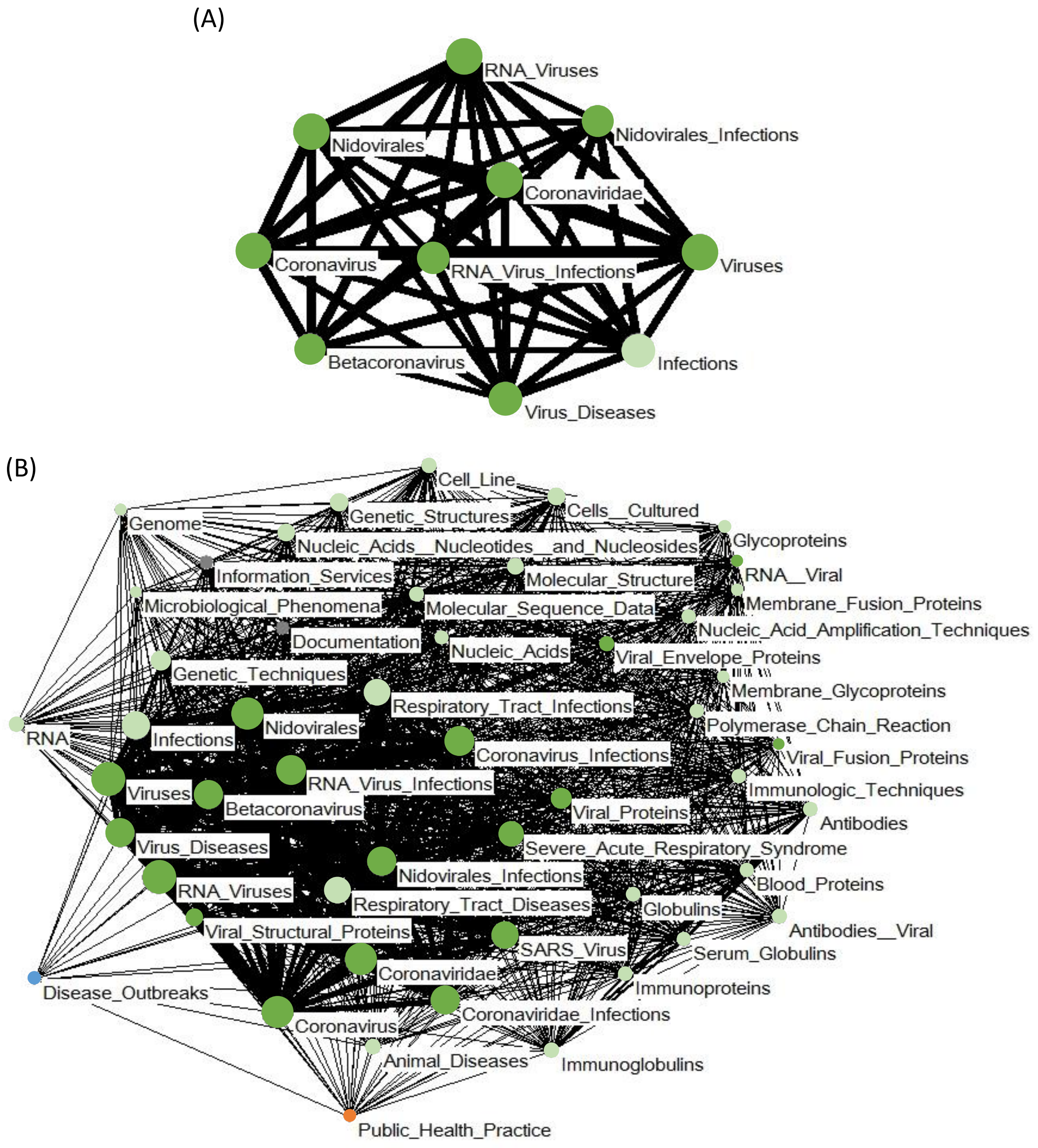

Supplementary Figure 2. The networks of the (A) top 10 and (B) top 50 unique keywords in 2003-2006. Only keywords that obtain links with other keywords are shown. The threshold for making edges was set at 10% of the number of keywords (selecting smaller sized nodes) linked by the edges. The node colors represent the fields related to the keywords (Green: virus and its infection, Light green: molecular biology, microbiology and immunology, Orange: health care and policy, Light orange: humanity and social issues, Blue: epidemiology, Grey: other issues).

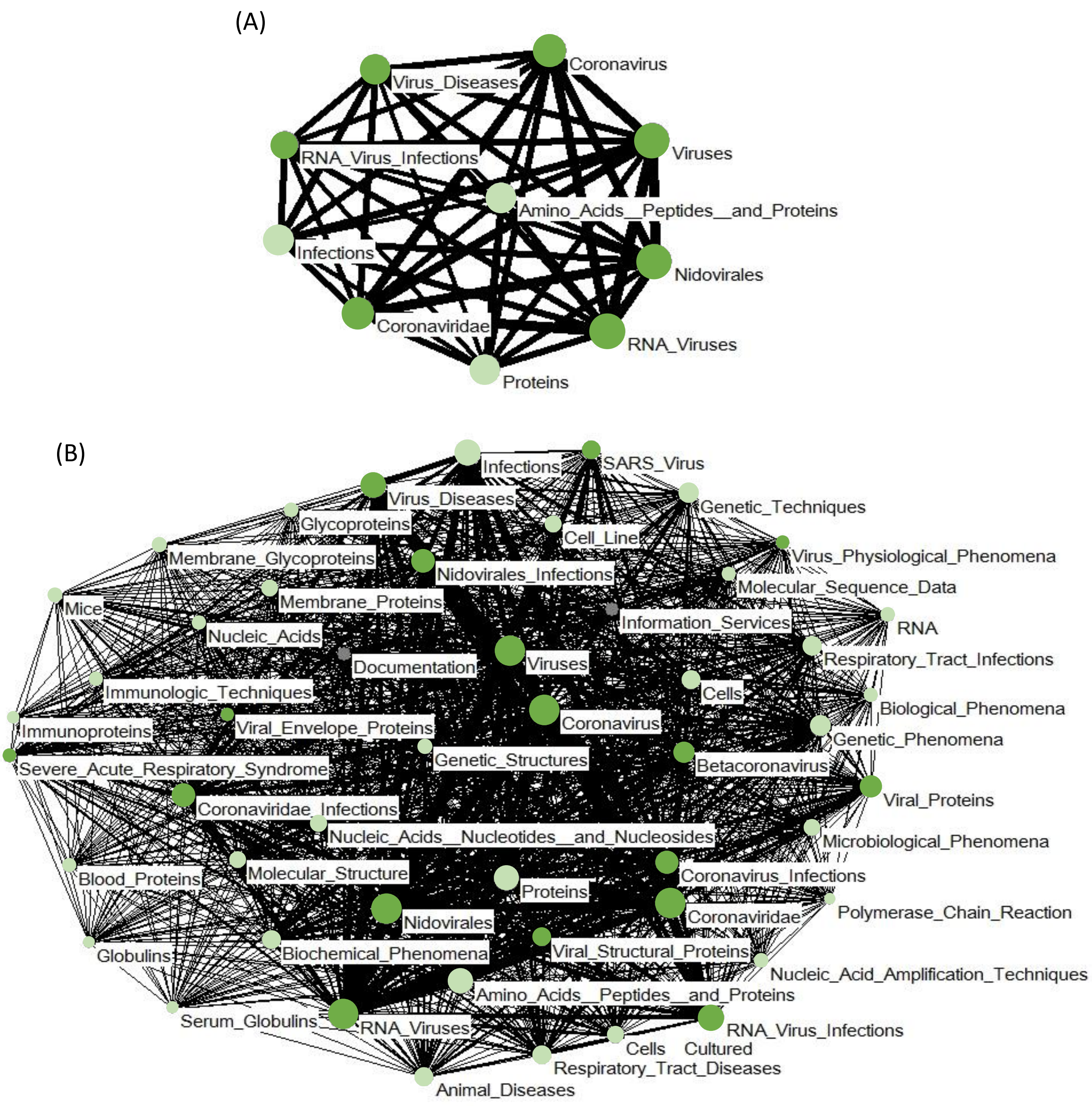

Supplementary Figure 3. The networks of the (A) top 10 and (B) top 50 unique keywords in 2007-2012. Only keywords that obtain links with other keywords are shown. The threshold for making edges was set at 10% of the number of keywords (selecting smaller sized nodes) linked by the edges. The node colors represent the fields related to the keywords (Green: virus and its infection, Light green: molecular biology, microbiology and immunology, Orange: health care and policy, Light orange: humanity and social issues, Blue: epidemiology, Grey: other issues).

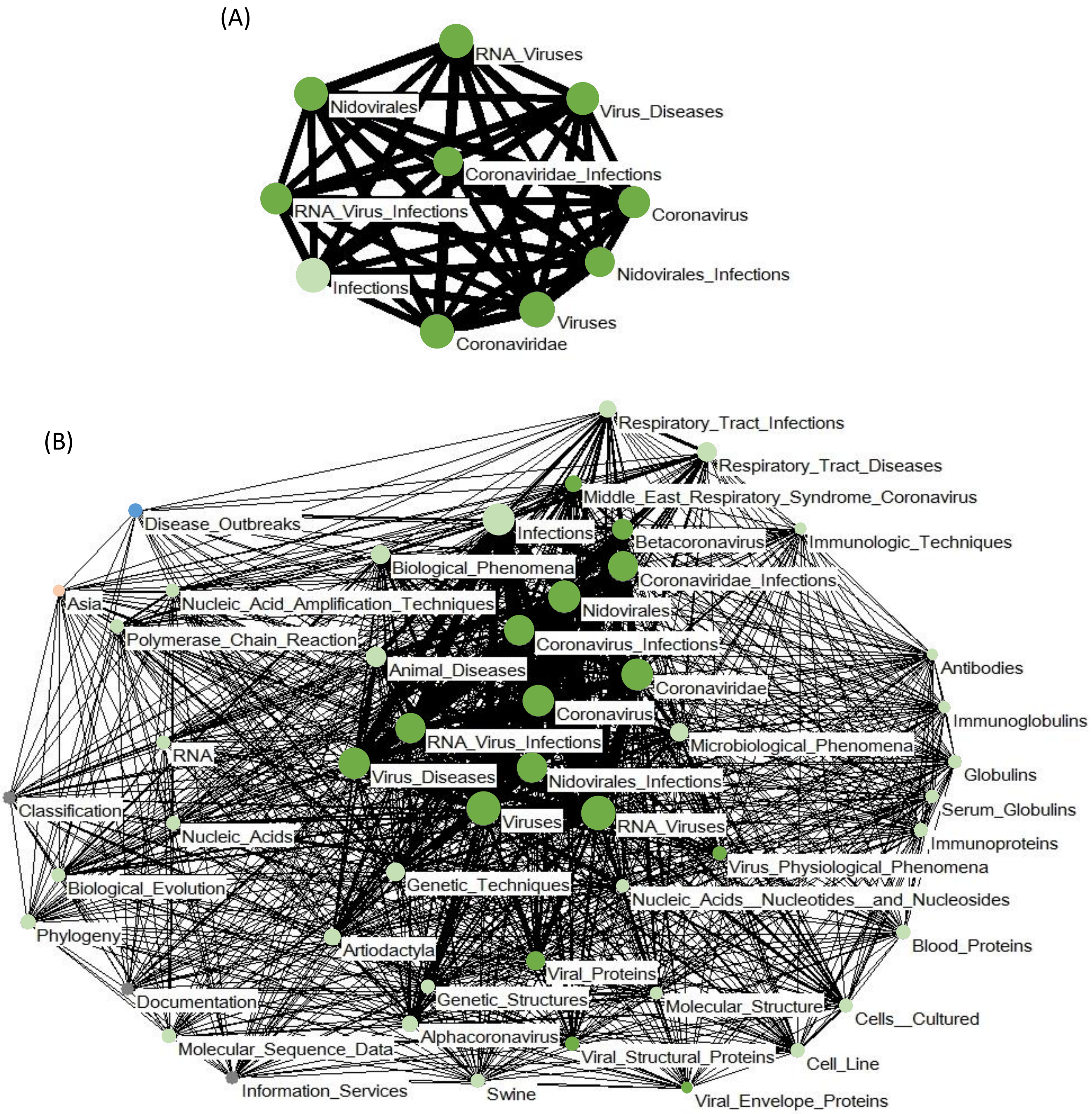

Supplementary Figure 4. The networks of the (A) top 10 and (B) top 50 unique keywords in 2013-2016. Only keywords that obtain links with other keywords are shown. The threshold for making edges was set at 10% of the number of keywords (selecting smaller sized nodes) linked by the edges. The node colors represent the fields related to the keywords (Green: virus and its infection, Light green: molecular biology, microbiology and immunology, Orange: health care and policy, Light orange: humanity and social issues, Blue: epidemiology, Grey: other issues).

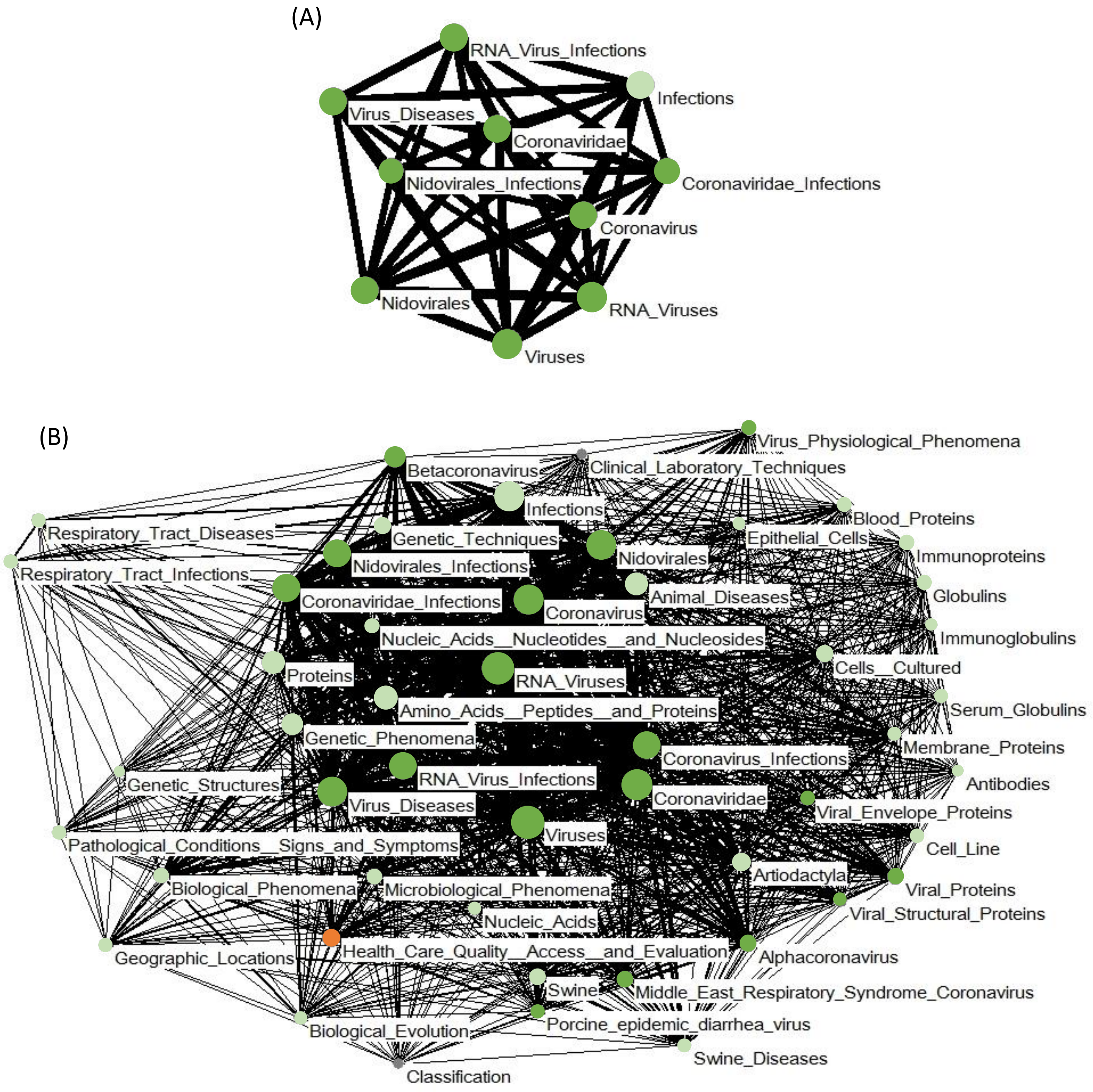

Supplementary Figure 5. The networks of the (A) top 10 and (B) top 50 unique keywords in 2017-2019. Only keywords that obtain links with other keywords are shown. The threshold for making edges was set at 10% of the number of keywords (selecting smaller sized nodes) linked by the edges. The node colors represent the fields related to the keywords (Green: virus and its infection, Light green: molecular biology, microbiology and immunology, Orange: health care and policy, Light orange: humanity and social issues, Blue: epidemiology, Grey: other issues).

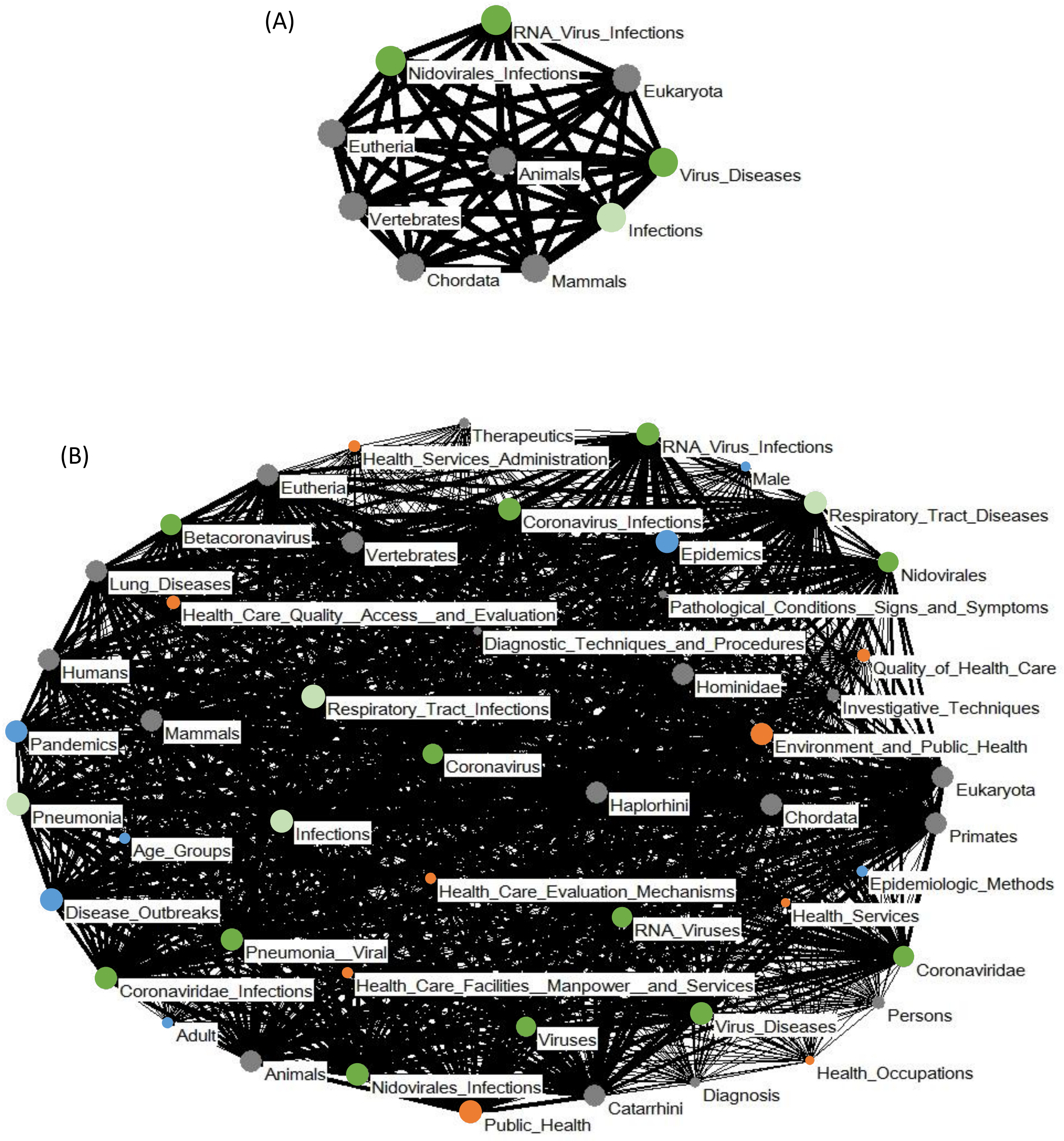

Supplementary Figure 6. The networks of the (A) top 10 and (B) top 50 unique keywords in 2020 till Aug. Only keywords that obtain links with other keywords are shown. The labels on the clusters represent the name of topics. The threshold for making edges was set as 10% of the number of keywords (selecting smaller sized nodes) linked by the edges. The node colors represent the fields related to the keywords (Green: virus and its infection, Light green: molecular biology, microbiology and immunology, Orange: health care and policy, Light orange: humanity and social issues, Blue: epidemiology, Grey: other issues).
